# Bone marrow toxicity associated with febantel administration in a dog: Case Report

**DOI:** 10.3389/fvets.2025.1557657

**Published:** 2025-04-07

**Authors:** Abbey Petronzio, David Carabetta, Audrey Koid

**Affiliations:** MSPCA-Angell, Boston, MA, United States

**Keywords:** fenbantal, bone marrow suppression, idiosyncratic adverse drug reaction, pancytopenia, drug toxicity and adverse effect

## Abstract

**Introduction:**

The objective of this case report is to describe a case of bone marrow suppression suspected to be secondary to febantel administration in a dog.

**Case summary:**

A 6-year-old neutered male Labrador Retriever was presented for lethargy and fever 15 days after receiving oral febantel. Pancytopenia was noted, and bone marrow sampling was consistent with hypoplasia suspected to be due to the febantel administration as no other toxic insults were identified. The dog made a full recovery in approximately 2 weeks after stopping the febantel.

**Discussion:**

This is the first report of suspected bone marrow toxicity secondary to febantel administration. Febantel is metabolized to fenbendazole, which has been documented as causing bone marrow toxicity in canines.

## 1 Introduction

Idiosyncratic drug reactions are uncommon toxicities that occur in patients receiving medications at recommended therapeutic doses (1). Idiosyncratic toxicity can be more difficult to predict in the general population compared to overdose. Idiosyncratic reactions to medications most often affect the liver, skin, and bone marrow in dogs and cats (1). When severe injuries to the bone marrow occur, bone marrow necrosis can develop and result in non-regenerative anemia, leukopenia, or thrombocytopenia (2).

Idiosyncratic bone marrow toxicity has been documented with the use of benzimidazole compounds commonly utilized in veterinary medicine to treat intestinal parasites. Albendazole has been shown to cause bone marrow toxicity in humans and has also been reported in dogs and cats (3–5). Fenbendazole is another benzimidazole anthelminthic that has been shown to cause bone marrow hypoplasia and bone marrow necrosis in various species (2, 6–9).

Febantel is an anti-parasitic medication used in small animals for the treatment of giardia when combined with pyrantel pamoate and praziquantel (brand name Drontal Plus^®^) (10). Febantel is metabolized to the benzimidazoles fenbendazole and oxfendazole, as shown *in vitro* studies and *in vivo* in cattle (11, 12).

To the best of these authors’ knowledge, no reports of bone marrow toxicity resulting from febantel use exist in dogs. However, fenbendazole has been reported to cause pancytopenia as a result of bone marrow toxicity (2, 6–9).

## 2 Case description

A 6-year-old, 35.6 kg, neutered male Labrador Retriever presented to a private specialty referral center in Massachusetts for evaluation of lethargy and 24 h of anorexia. He was adopted from a Labrador rescue 5 years ago and was treated for heartworm the same year. He was historically positive for Anaplasma antibodies on the SNAP 4DX Test (IDEXX^®^) and was treated 5 years ago with a 30-day course of doxycycline. He has continued to test positive on SNAP 4DX Tests (IDEXX^®^).

Another dog in the household was diagnosed with giardiasis via point of care antigen test 14 days prior, and although this patient was never symptomatic nor fecal antigen positive, he was empirically treated as well. He was prescribed Drontal Plus^®^ for dogs 45 lbs. and greater (1.5 tablets contain 5.7 mg/kg of praziquantel, 5.7 mg/kg of pyrantel pamoate, and 28.6 mg/kg of febantel total) orally once daily for 5 days. The owner reported decreased appetite during 5 days of receiving Drontal Plus^®^. This progressed to anorexia 24 h prior to presentation (approximately 15 days after starting the course of Drontal Plus^®^).

On presentation, the dog had a heart rate of 84/min, slightly elevated respirations with a soft dry cough (32/min), and a rectal temperature of 40.8°C (105.6°F). He was also noted to be nauseous, mildly dehydrated, and weak. Blood samples for a complete blood count (CBC), blood chemistry, urinalysis, thyroid panel, and PCR testing for tick-borne illnesses, as well as the SNAP 4DX Plus Test (IDEXX^®^), were taken and submitted upon admission.

He was started on intravenous fluid therapy with Lactated Ringer’s solution at 40 mL/kg/day, maropitant 1 mg/kg IV once a day, ondansetron 1 mg/kg IV every 8 h, and gabapentin at 300 mg orally every 8 h. Thoracic radiographs were taken overnight given the soft cough noted. Transient fluid in the distal esophagus was noted; otherwise, the thoracic radiographs were normal as interpreted by a board-certified radiologist.

The CBC returned showing severe leukopenia (white cell count of 0.3 k/uL; reference interval (RI): 5.6–15.1 k/uL), thrombocytopenia (39 k/uL automated count; estimate 40–60 k/uL on manual count; RI: 161–513 k/uL), and a normal red blood cell count (hematocrit of 41.4%; RI: 39.9–58.2%) (Table 1). A pathologist review of the CBC listed drug toxicity as a possible differential and a bone marrow evaluation was recommended if no further cause of leukopenia or thrombocytopenia was found. At this point, ampicillin/sulbactam at 33 mg/kg of ampicillin IV and 16.5 mg/kg of sulbactam IV every 6 h was added due to the risk of sepsis with severe leukopenia.

**Table 1:** Complete blood count results from day 1 to 1 month post discharge.

Parameter	Day 1	Day 3	Day 4	Day 5	Day 12	Day 35	Reference range
WBC (k/uL)	*0.3*	*1.2*	*3.2*	*5.4*	13.4	8.1	5.6–15.1
RBC (10^6^/uL)	6.00	*4.82*	*4.55*	*5.40*	6.20	7.13	5.75–8.63
Hgb (g/dL)	14.6	*11.5*	*10.6*	*12.8*	15.6	17.3	14.0–21.1
Hct (%)	41.4	*32.9*	*31.1*	*36.8*	42.4	48.5	39.9–58.2
MCV (fL)	69.0	68.3	68.4	68.1	68.4	68.0	63.8–72.9
MCH (pg)	24.3	23.9	23.3	23.7	25.2	24.3	22.3–26.5
MCHC (g/dL)	35.3	35.0	34.1	34.8	36.8	35.7	33.8–37.5
Platelets (k/uL)	*39*	*60*	*105–120*	*133*	335	223	161–513
Reticulocyte count (k/uL)	23.4	20.2	*11.4*	18.9	28.5	46.3	13.8–125.1; Absolute count >80 consistent with canine regeneration
Corrected reticulocyte percentage (%)	0.3	0.3	0.2	0.3	0.4	0.7	>1.0% consistent with canine regeneration
Segmented neutrophils (k/uL)	Unable to be determined	*0.5*	*1.6*	*3.5*	*10.2*	6.3	3.1–9.8
Band neutrophils (k/uL)	Unable to be determined	0.0	0.0	0.0	None	None	
Lymphocytes (k/uL)	Unable to be determined	*0.7*	1.2	1.6	2.4	0.9	1.2–5.4
Monocytes (k/uL)	Unable to be determined	*0.0*	0.2	0.3	0.4	0.4	0.2–0.7
Eosinophils (k/uL)	Unable to be determined	0.0	0.0	0.1	0.3	0.5	0.0–1.5
Morphologic changes		1+ toxic change	Large platelets, 1+ toxic change	Large platelets, slight toxic change, 3 NRBCs/100 WBCs		3 NRBCs/100 WBCs	

Values in italics are outside of their reference ranges.

The initial blood chemistry showed an elevated alkaline phosphatase (ALP) at 196 U/L (RI: 12–116 U/L), an elevated total bilirubin at 0.4 mg/dL (RI: 0.0–0.3 mg/dL), and a normal total protein at 6.4 g/dL (RI: 5.4–7.0 g/dL). A SNAP 4DX Plus Test (IDEXX^®^) was positive for Anaplasma (a historic finding). A Canine FastPanel PCR Tick Borne Panel (Antech^®^) returned 4 days after presentation, negative for *Anaplasma phagocytophilum*, *Anaplasma platys*, Babesia canis, Babesia spp. (non-canis), *Bartonella henselae*, *Bartonella vinsonii*, *Ehrlichia Canis*, *Ehrlichia* spp., *Mycoplasma haemocanis*/*hemoparvum*, *Neorickettsia risticii*, and *Rickettsia rickettsii*, indicating no active infection was present. Low free thyroxine and total thyroxine were also found but suspected to be due to euthyroid sick syndrome (13–15). A urinalysis showed a urine specific gravity of 1.036, pH 7, and 2+ protein, and canine pancreatic lipase was highly normal at 203 ug/L (RI: 3–100 ug/L). An abdominal ultrasound showed a normal abdomen. Doxycycline was added at 200 mg orally every 12 h given the persistent fever.

On day 3 of hospitalization, the dog’s fever resolved, but he was still anorexic. A repeat CBC showed improved leukopenia (1.3 k/uL; RI: 5.6–15.1 k/uL), static to improved thrombocytopenia (60 k/uL; RI: 161–513 k/uL), and new non-regenerative anemia (32.9%; RI: 39.9–58.2%) with a corrected canine reticulocyte percentage of 0.3% and an absolute reticulocyte count of 20.2 k/uL (canine regeneration consistent with the corrected percentage of >1.0 and absolute count of >80 K/uL). The patient had toxic changes and band neutrophils. A retest of liver chemistry was performed with a static ALP elevation (196 U/L; RI: 12–116 U/L), slightly increased hyperbilirubinemia (0.5 mg/dL; RI: 0.0–0.3 mg/dL), mild hypoalbuminemia (3.0 g/dL; RI: 3.1–4.2 g/dL), and a decreased total protein of 4.9 g/dL (RI: 5.4–7.0 g/dL).

**Figure 1:**
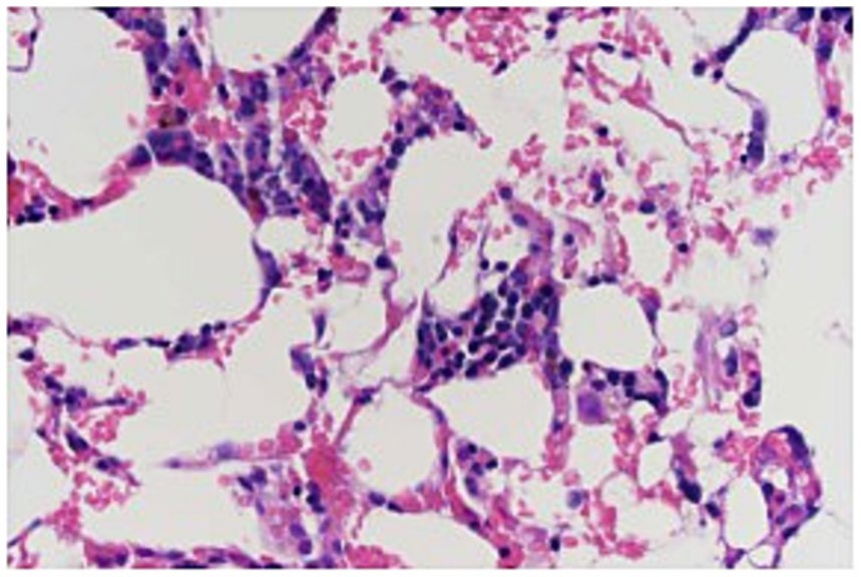
Bone marrow biopsy specimen from a 6-year-old dog with pancytopenia following febantel therapy. Megakaryocytes are present with normal morphology. Myeloid precursors markedly decreased, but too few were present to evaluate maturation. The estimated M:E ratio was approximately 1:4. Erythroid precursors decreased and exhibited orderly maturation (Hematoxylin and eosin stain; 400×).

The dog was sedated with midazolam 0.2 mg/kg IV, ketamine 1 mg/kg IV, methadone 0.2 mg/kg IV, and propofol 2.4 mg/kg to maintain sedation. Bone marrow aspirates and core biopsies were taken from the right humerus. The initial cytology of the bone marrow showed marked marrow hypoplasia. However, only two very small poorly cellular spicules were obtained. No megakaryocytes were identified in the spicules, and myeloid precursors were markedly reduced (although too few were present to evaluate for maturation). Erythroid precursors were also moderately reduced. The histology of the core biopsy was also consistent with marked marrow hypoplasia (Figure 1). The needle core biopsy only contained <10% hematopoietic cell lines. Megakaryocytes were rare but exhibited normal morphology. Myeloid precursors were markedly decreased, but too few were present to evaluate maturation. The estimated M:E ratio was approximately 1:4. Erythroid precursors were decreased and exhibited orderly maturation. Given these findings, an idiosyncratic reaction to Drontal Plus^®^ was considered to be the most likely cause of pancytopenia.

A nasogastric tube was placed and feedings with Royal Canin^®^ Gastrointestinal Low Fat Liquid diet started at ¼ resting energy requirement (RER) or 70 mL via nasogastric tube every 6 h given the persistent anorexia. He was also started on methadone at 0.2 mg/kg IV every 6 h post bone marrow aspirates and core biopsy.

On day 4, the dog’s leukopenia (3.2 k/uL; RI: 5.6–15.1 k/uL) and thrombocytopenia (105–120 k/uL on manual count; RI: 161–513 k/uL) both improved. However, his anemia was mildly worse at 31.3% (RI: 39.9–58.2%), with a corrected reticulocyte percentage of 0.2% and an absolute reticulocyte count of 11.4. He started eating on his own and had a normal temperature.

On day 5, leukopenia improved to 5.4 k/uL (RI: 5.6–15.1 k/uL), thrombocytopenia to 133 k/uL (automated count; RI: 161–513 k/uL), and anemia to 36.8% (RI: 39.9–58.2%). A reassessment of his liver chemistry showed that his ALP had increased to 827 U/L (RI: 12–116 U/L), but his hyperbilirubinemia improved to 0.2 g/dL (RI: 0.0–0.3 mg/dL) along with his hypoalbuminemia (3.1 g/dL; RI: 3.1–4.2 g/dL). At this stage, he was discharged on 200 mg doxycycline orally every 12 h until the tick PCR results were obtained.

On day 12 (1 week after being discharged from the hospital), the dog returned for another CBC and blood chemistry tests. His leukopenia (13.4 k/uL; RI: 5.6–15.1 K/uL), thrombocytopenia (335 k/uL on automated count; RI: 161–513 K/uL), and anemia (42.2%; RI: 39.9–58.2%) had all improved. His ALP improved to 581 U/L (RI: 12–116 U/L), but it remained high. He also had a newly elevated ALT at 119 U/L (RI: 22–74 U/L). The owner reported that he was clinically normal and no longer on oral medications aside for routine preventatives.

One month later, he was seen by his primary care veterinarian, and both his CBC and blood chemistry were normal.

## 3 Discussion

Benzimidazole compounds are widely used in veterinary medicine as effective anthelminthics. Their primary mechanism of action is interfering with microtubule organization inside the parasite cell. This leads to the inability of the parasite to absorb nutrients and eliminate waste, resulting in parasite death (12). Bone marrow suppression has been reported in benzimidazole agents, such as fenbendazole and albendazole (2–9).

Febantel is available in combination with pyrantel pamoate and praziquantel in the product Drontal Plus^®^. Drontal Plus^®^ has been used to treat giardiasis at dosages of over 3–5 days (16). The dog in this case was treated with the proper dosage for presumptive giardiasis (28 mg/kg by mouth once a day for 5 days), although a diagnosis was never made (10).

Pancytopenia can be caused by two broad categories: decreased hematopoietic cell production or increased hematopoietic cell destruction (17). Given the bone marrow aspirate and core biopsy were suggestive of hypoplastic marrow, the former was considered the cause of the pancytopenia in this case. Hemodilution could also be considered as the cause of anemia in this case, as the total protein also decreased from 6.4 g/dL on day 1 of hospitalization to 4.9 g/dL when anemia was noted.

Bone marrow hypoplasia occurs secondary to destruction or genetic defects in stem cells, an altered microenvironment within the marrow, or dysregulation of cell production (18, 19). In drug-induced bone marrow suppression, the mechanism of action is often not determined. Given the life span of neutrophils and platelets (1 to 4 days and 8 to 10 days, respectively), leukopenia and thrombocytopenia typically occur within 2 weeks of the initial bone marrow injury (17). In this case, the dog’s clinical signs and subsequently diagnosed leukopenia and thrombocytopenia occurred approximately 10 days following the cessation of Drontal Plus^®^ therapy, fitting the timeline for drug-induced bone marrow toxicity. However, it must be noted that the bone marrow hypoplasia was a presumed diagnosis as the bone marrow core biopsy and aspirates were of poor cellularity.

Once the drug has been eliminated from the body, stem cells will repopulate the marrow progenitor cells, and the cytopenia begins to resolve (24). This usually occurs approximately 1 to 2 weeks after the cessation of the drug (25). Recovery is considered complete within 21 days (17). Again, the dog in this case showed complete resolution of his pancytopenia 1 month after his hospitalization.

Based on accepted grading schemes in human medicine, this case can be classified as a possible or probably adverse drug reaction (20). The laboratory findings of pancytopenia occurred at a reasonable time in relation to the drug administration. However, the pancytopenia and clinical signs could also be explained by underlying concurrent diseases, such as Anaplasma infection in this case.

*Anaplasma phagocytophilum* has been shown to cause pancytopenia in humans (human granulocytic anaplasmosis) rarely; however, it is more likely to cause thrombocytopenia in dogs (21, 22). The dog described in this case was negative for both *Anaplasma phagocytophilum* and *Anaplasma platys* on PCR. It is possible that this was a false-negative result; however, no morulae in the neutrophils were seen on a peripheral blood smear. It is also possible that a concurrent Anaplasmosis infection could have pre-disposed this dog to an adverse drug reaction and resulted in greater toxicosis (23).

Other differentials for bone marrow hypoplasia include idiopathic and infectious causes. Infectious diseases in dogs causing bone marrow hypoplasia include canine parvovirus infection, *Ehrlichia canis* infection, *Babesiosis* infection, bacterial septicemia, and endotoxemia (17). The dog in this case was vaccinated for parvovirus and had no reported vomiting or diarrhea. A tick PCR panel was negative for any Ehrlichia or Babesia infections. No signs of septicemia or endotoxemia were found; however, it is important to note that neither condition can be completely excluded. An idiopathic cause is considered unlikely given the drug history in this case.

It is suspected that this was a case of idiosyncratic drug reaction given that Drontal Plus^®^ was given at therapeutic dosages. Fenbendazole, an active metabolite of febantel, is known to cause bone marrow suppression. No other drug exposure was reported, and no other causes of pancytopenia were found.

## Data Availability

The original contributions presented in the study are included in the article/supplementary material, further inquiries can be directed to the corresponding author.
